# Ecological effect life cycle assessment of house buildings based on emergy footprint model

**DOI:** 10.1038/s41598-023-43501-3

**Published:** 2023-09-30

**Authors:** Mengyang He, Yang Wang, Haotian Ma

**Affiliations:** Institute of Economics and Management, Shanghai University of Electric Power, Shanghai, 201306 China

**Keywords:** Civil engineering, Energy efficiency, Environmental impact, Environmental impact, Ecological modelling

## Abstract

Construction is an important sector for climate action. The construction, operation and maintenance, demolition and disposal stages of house buildings consume many resources and have a significant impact on society, the economy and the environment. To assess such efforts, we propose the emergy footprint model of house buildings, which can quantitatively analyse the ecological effect in the house buildings life cycle. The research shows the following. China’s ecological efficiency of the housing sector is characterized by improvement. In the house building fifty-year life cycle, the emergy footprint of the operation and maintenance stage is the largest (75.92%), followed by the construction stage (21.95%), but the emergy footprint intensity of the latter is 4.82 times that of the former. Reducing energy consumption and carbon dioxide emissions in the operation and maintenance stage is the key to reducing the life cycle emergy footprint of house buildings. The ecological impact coefficient of house buildings is negatively exponentially correlated with their service life. It reaches ecological break-even when the service period of the house building is equal to 36.73 years. If the house building is demolished after less than nine years of service, the impact is extremely unfavourable.

## Introduction

Buildings and construction activities consume a large amount of resources and have a significant impact on society, the economy and the environment. In 2019, the building and construction sector was connected to 38% of total global energy-related carbon dioxide emissions, with operational emissions of approximately 10 Gt CO_2_ or 28% and emissions from the construction industry representing an additional 10%^1^. In 2021, the energy demand of buildings increased by approximately 4% from 2020 to 4.61 billion tons^1^. The buildings and construction industry is an important sector for climate action.

The energy consumption of buildings and the construction sector accounts for approximately 34%^2^ of the total energy consumption of China. China is in a historical period of rapid industrialization, urbanization and new rural construction. The urbanization rate of China rose from 10.64% in 1949 to 64.72% in 2021^3^. In this process, more than four billion square metres of house buildings are completed every year in China, which consumes many resources. For example, in 2020, the output of steel and cement in China was 1.32 billion tons^4^ and 2.38 billion tons^5^, respectively, while China's buildings and construction industry consumed 989 million tons of steel and 2.32 billion tons of cement in 2020^6^, accounting for 74.64% and 97.48% of China’s total output in that year, respectively. China's carbon dioxide emissions will peak before 2030 and it is striving to achieve carbon neutrality before 2060^7^. Energy saving, emission reduction, and implementation of low-carbon and ecological buildings in this sector are important aspects for China to achieve its greenhouse gas-independent action goals.

The environmental impact assessment of buildings is a multifactor and multiobjective assessment of social-economic-ecological complex systems, and it has been given increasing attention^8^ and has been developed from single-stage evaluation^9^ to life cycle assessment^10,11^. Multifactor evaluation of the life cycle can enable the environmental impact assessment of multiple environmental impact factors on the life cycle of buildings^12,13^. According to the number of assessment factors, the quantitative assessment method of the impact of buildings on the environment can be divided into single-factor and multifactor assessment methods. The single-factor assessment method analyses the environmental impact of a certain factor, such as building materials^14,15^ and energy consumption^16^. This method can compare and analyse the impact of the same building in different stages or different buildings. However, different impact factors have different dimensions, such as energy consumption, building materials and carbon dioxide emissions, and the single-factor assessment method is not conducive to the horizontal comparison of different environmental impact factors. The ecological footprint model and emergy analysis theory can be used for multifactor quantitative evaluation with different dimensions^17^.

From the perspective of biological productivity, the ecological footprint Model^18^ uses yield factors and equilibrium factors to calculate the ecological capacity and ecological footprint, judges the ecological profit and loss, and quantitatively measures the comprehensive impact of the study object on the environment. Emergy analysis theory^19^ transforms different kinds, different levels and incomparable energy flows, material flows and currency flows of the social-economic-ecological system into emergy, which have the same dimension. Emergy analysis theory provides an energetics base for quantitatively assessing the products and services of social-economic-ecological compound systems.

In recent years, there have been some studies and good applications of emergy analysis theory^20,21^ and ecological footprint model^22^ in terms of the quantitative evaluation of the environmental impact of house buildings. The emergy footprint model combines the advantages of the ecological footprint model and emergy analysis theory, and thus has developed into a new quantitative evaluation method of ecological environmental impact^23^. For example, the emergy footprint model constructed in the study realizes the quantitative evaluation of the ecological effects of the *Three Gorges Project* during the construction and operation stages^24^. The dynamic decomposition and regional differences were studied in *Yangtze River Delta* cities with an emergy footprint model^25^. The emergy ecological footprint model has been applied to the security and sustainability development of water resources^26^. The water ecological footprint is analysed with emergy analysis and spatial autocorrelation analysis to quantify and analyse the human demand for water resources and the available supply of water resource systems^27^. Based on the emergy analysis, the ecological footprint of a wind farm is estimated, which includes wind turbine production and transportation, construction, operation and maintenance, and demolition during the life cycle^28^. Based on the emergy ecological footprint model, the ecological status of logistics ports is analysed, and some suggestions for developing green logistics ports are put forwards^29^.

The emergy footprint model can solve two major problems at present, namely, the unified evaluation among different environmental factors and the unified evaluation among social, economic and ecological environmental factors^24^. However, multifactor ecological effect life cycle assessments of house buildings based on the emergy footprint model have not been appropriately carried out.

According to the *China Statistical Yearbook on Construction*^6^, the building and construction industry is divided into four subindustries, namely, the house building industry, civil engineering construction industry, building installation industry, building decoration and other construction industry. In this paper, house buildings will be taken as the research object, which includes steel, concrete, masonry and wooden house buildings. The emergy footprint model of house buildings will be constructed by organically combining the emergy analysis theory and the ecological footprint model. In the process of model construction, the following factors will be analysed; namely, the resource consumption and environmental emissions in the construction, operation and maintenance, demolition and disposal stages according to the characteristics of the social, economic and ecological environment impact of house buildings in the life cycle. This model is expected to realize the quantitative evaluation of the environmental impact of the building life cycle.

## Methodology

### Emergy analysis system diagram

**Figure 1:**
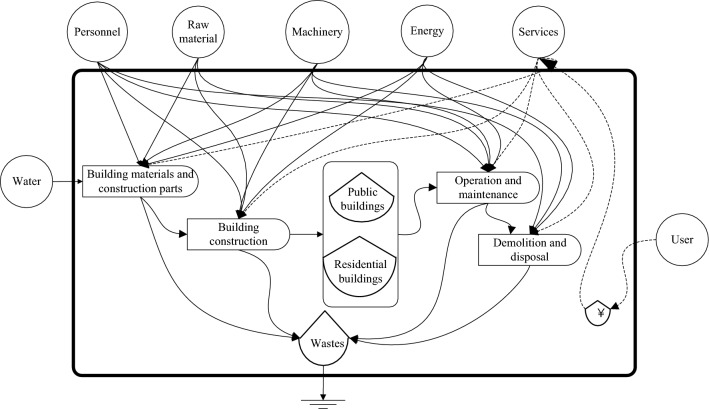
Emergy analysis system diagram of house building life cycle assessment.

The construction, operation and maintenance, demolition and disposal of house buildings need to consume various resources and emit environmental impacts. Therefore, it is necessary to erect a quantitative evaluation model of the environmental impact of house buildings from the perspective of the life cycle. The life cycle of house buildings includes four stages: production of building materials and construction parts, construction, operation and maintenance, and demolition and disposal. The house building sector mainly provides residential and public building products. According to emergy analysis theory, life cycle assessment (LCA) and input‒output (I–O) theory, the emergy analysis system diagram of the life cycle assessment of house buildings is shown in Fig. 1.

### Emergy footprint model

**Figure 2:**
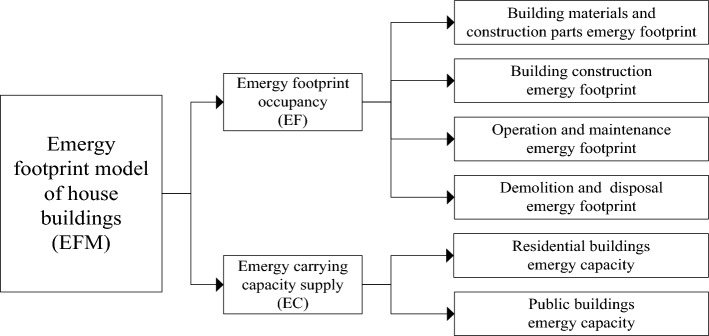
Emergy footprint model of house buildings.

The emergy footprint model of house buildings includes emergy footprint occupancy and emergy carrying capacity supply, as shown in Fig. 2. The emergy footprint occupancy account consists of four parts, namely, the building materials and construction parts emergy footprint, building construction emergy footprint, operation and maintenance emergy footprint, and demolition and disposal emergy footprint. The emergy carrying capacity supply account consists of residential and public building carrying capacity supply two parts.

#### Emergy and emergy density

Emergy is defined as the energy of one type required in transformations to generate a flow or storage, which can be calculated using Eq. (1)^19^.

1 $$E=Q\cdot \tau ,$$

where *E* is the emergy of a certain resource to be consumed or a kind of environmental emission of the house buildings, *Q* is the quantity of an item, and *τ* is the transformity of this item.

According to the ecological footprint model^18^, the unit is the global hectare (hm^2^) of ecological footprint and ecological carrying capacity. In emergy analysis theory, the unit of emergy is solar energy joule (sej). This requires a bridge to convert emergy into “bio-productive land area” (hm^2^). Therefore, the concept of emergy density will be introduced.

Emergy density is defined as the ratio of the total emergy used by a region to its area and can be expressed as Ref.^23^

2 $$D={E}_{t}/A,$$

where *D* is the emergy density (sej/hm^2^), *E*_*t*_ is the total emergy use of a region (sej) and *A* is the area of this region (hm^2^).

Emergy density reflects the economic development level or the development intensity of the region and can be used to evaluate the emergy intensity and strength of this region. The greater the emergy density of a region is, the more developed the economy or the greater the development intensity is.

The total emergy entering China’s social and economic system is 2.40 × 10^25^ sej^24^. The construction land of cities, towns, villages, industries and mines in China increased from 2.69 × 10^7^ hm^2^ in 2011 to 3.53 × 10^7^ hm^2^ in 2020^3^, with a mean of 3.05 × 10^7^ hm^2^. According to Eq. (2), the average emergy density of construction land in China is 7.87 × 10^17^ sej/hm^2^.

#### Emergy footprint account

The emergy input in the house building life cycle can be converted into an emergy footprint, and the completed area provided by house buildings can be converted into emergy capacity with the emergy density.

1. Various resources have the attribute of occupying the service function of social ecosystems, such as construction land, personnel, materials, machinery and equipment, energy, and wastes discharged into the environment. The emergy footprint of house buildings (EF) refers to the area of bio-productive land converted from various resources invested in the house building life cycle, which is formulated as
  3 $$EF=\frac{1}{D}\sum_{i=1}^{\mathrm{n}}\left({Q}_{i}\times {\tau }_{i}\right),$$
  where ${Q}_{i}$ is the amount of input or environmental emission of the i*th* item during the life cycle of the house buildings. For example, all kinds of construction materials, parts and land, personnel, waste and greenhouse gases emitted during the construction phase; the energy and maintenance material consumption, personnel and services invested, greenhouse gas emissions and other wastes in the operation and maintenance stage; and the personnel and services invested, energy consumption, greenhouse gas emissions and other wastes in the demolition and disposal stage.${\tau }_{i}$ is the transformity of the i*th* item.
2. The emergy capacity of house buildings (EC) refers to the area of bio-productive land converted from the completed area of all kinds of house buildings in society through building construction activities. It is equal to the sum of all kinds of house buildings with the completed area times their actual service life times the corresponding transformity and then divided by the emergy density of construction land, that is:
  4 $$EC=\frac{1}{D}\sum_{k=1}^{\mathrm{m}}\left[\left({S}_{k}\times {L}_{k}\right)\times {\tau }_{k}\right],$$
  where ${S}_{k}$ is the completed area of the k*th* item house buildings (hm^2^), ${L}_{k}$ is the actual service life of the k*th* item house buildings (year), and ${\tau }_{k}$ is the transformity of the k*th* item house buildings (sej/hm^2^.year). Among them, the transformity of house buildings (${\tau }_{h}$) refers to the average annual total emergy input per square unit area during the normal service life of house buildings. It is equal to the total emergy (${E}_{h}$) of the house building life cycle (${Y}_{r}$) divided by the building area (*A*) and divided by the normal service life (*n*), namely:
  5 $${\tau }_{h}=\frac{{E}_{h}}{A\times n}=\frac{{E}_{h}}{A\times \left({Y}_{r}-T\right)},$$
  where *T* is the time required for the land occupied by the building from the acquisition of land use rights, preparation before construction, completion of construction and decoration to normal service.

#### Ecological effect analysis

The impact of house buildings on the economy, society and ecological environment can be evaluated by the ecological profit and loss and the ecological impact coefficient.

1. Ecological profit and loss (EP) reflects that the impact of house buildings on the social, economic and ecological environment is generally positive or negative. It is equal to the difference between the total emergy capacity and the total emergy footprint of house buildings, namely:
  6 $$EP=EC-EF.$$
  The impact of house buildings on the social, economic and ecological environment is ecological surplus when EP is greater than zero; it is an ecological deficit when EP is less than zero, and it is just ecological balance when EP equals zero.
2. The ecological impact coefficient (γ) is a nondimensional parameter that reflects the impact of house buildings on the social, economic and ecological environment in the whole life cycle and is equal to the ratio of the emergy footprint and emergy capacity of house buildings, that is,
  7 $$\gamma =EF/EC.$$

The smaller γ of the house buildings is, the more beneficial it is to the sustainable development of society, economy and ecological environment. In contrast, it is not conducive to sustainable development. According to the magnitude of the ecological impact coefficient, the comprehensive impact level of house buildings can be divided into five levels (Table 1).

**Table 1:** Comprehensive impact grading table of house buildings on society, economy and environment.

Ecological impact coefficient	γ < 0.95	0.95 ≤ γ < 1	γ = 1	1 < γ < 2	γ ≥ 2
Impact level	Positive (I)	More favourable (II)	Break-even (III)	Unfavourable (IV)	Extremely unfavourable (V)

## Data collection and processing

In this paper, the life cycle of house buildings is divided into the construction, operation, maintenance, demolition and disposal stages. The emergy footprint model constructed in this paper is used for the quantitative evaluation of the whole life cycle. This paper mainly uses the official statistical data of the "China Statistical Yearbook" and "China Construction Industry Statistical Yearbook" of the National Bureau of Statistics of China. These data are authoritative and reliable. Excel software was used for evaluation.

### Construction stage

The consumption of steel, cement, wood, glass, aluminium and energy and the output value of construction and house buildings in China over the years can be obtained according to the *China Statistical Yearbook on Construction*^6^, and the proportion of the output value of the house buildings to the construction industry in the output value of the construction industry can be calculated (Appendix Table 1). Based on this ratio, for example, 61.33% in 2020, the consumption of steel, cement, wood, glass, aluminium, energy and employed staff of the house buildings over the years, as well as the main resource consumption per construction area square metres of the house buildings over the years, can be estimated (Table 2).

**Table 2:** Main resource consumption of construction area per square metre of house building.

Item	Unit	2011	2012	2013	2014	2015	2016	2017	2018	2019	2020	Average
Steel	t	5.05E − 02	6.21E − 02	4.44E − 02	5.19E − 02	4.22E − 02	4.54E − 02	4.51E − 02	4.17E − 02	5.05E − 02	4.37E − 02	4.78E − 02
Cement	t	2.17E − 01	2.53E − 01	1.43E − 01	1.44E − 01	1.10E − 01	1.14E − 01	1.20E − 01	1.13E − 01	1.14E − 01	1.02E − 01	1.43E − 01
Wood	m^3^	1.80E − 02	2.66E − 02	1.80E − 02	2.09E − 02	2.00E − 02	2.41E − 02	2.40E − 02	2.27E − 02	2.32E − 02	2.38E − 02	2.21E − 02
Glass	t	5.26E − 04	8.44E − 04	1.85E − 03	8.09E − 04	5.25E − 04	4.96E − 04	5.54E − 04	5.08E − 04	5.29E − 04	4.12E − 04	7.05E − 04
Aluminium	t	2.91E − 03	4.33E − 03	3.08E − 03	3.33E − 03	3.10E − 03	2.99E − 03	2.91E − 03	2.98E − 03	6.11E − 03	2.72E − 03	3.45E − 03
Energy	tce	4.47E − 03	4.19E − 03	4.19E − 03	4.14E − 03	4.23E − 03	4.29E − 03	4.57E − 03	4.05E − 03	4.30E − 03	4.11E − 03	4.25E − 03
Employed staff	person	3.82E − 03	3.14E − 03	3.00E − 03	3.06E − 03	3.07E − 03	3.08E − 03	3.28E − 03	2.78E − 03	2.92E − 03	2.37E − 03	3.05E − 03

There are some differences in the consumption of materials such as sand, stone and water for concrete with different strengths. Taking C35 concrete used in most structures as an example, the cement, sand, stone and water consumption for making one cubic metre concrete on site is 424, 581, 1179 and 195 kg, respectively. The average consumption of cement per square metre of house building construction area is 0.143 tons (Table 2). According to the above concrete mix ratio, the average consumption of sand, stone and water per square metre of house building construction area is 0.196, 0.398 and 0.07 tons, respectively. For pouring concrete per cubic metre, 1700–2400 L of water will be needed. The mean is taken in the calculation, namely, 2050 L. In addition, the imbalance coefficient is taken as 1.5^30^. The total water consumption per square metre construction area of a house building is 1.11 L.

According to Ref.^31^, the consumption of bricks, ceramics and other materials per square construction area in the construction stage, as well as the emission of environmental impact substances such as COD, NH_3_-N, SO_2_ and NO_x,_ can be calculated. According to Ref.^32^, the emissions of CO_2_ and CO can be calculated, and the results are shown in Table 3. The floor space of the house building is equal to the gross completed area calculated for the floor space ratio divided by the floor space ratio. In the calculation, the floor space ratio is the average floor space ratio of high-rise residential buildings, namely, 2.5, so the floor space of each square metre of house building is 0.4 square metres.

**Table 3:** Other resource consumption and environmental impact emissions per square metre construction area in the construction stage.

Item	Sand & gravel	Water	Brick	Ceramic	COD	NH_3_-N	SO_2_	NO_x_	CO_2_	CO
Quantity (t)	5.94E − 01	1.11E + 00	1.06E − 01	1.90E − 02	3.86E − 04	2.51E − 06	6.12E − 04	6.21E − 04	8.07E − 02	3.33E − 03

### Operation and maintenance stage

China has a vast territory and complex terrain, and the climate varies greatly from place to place. The constraint values of building energy consumption indicators in different climate zones are different, and the energy consumption of house building operation and maintenance is also different. In 2018, the total building area of China was approximately 60.1 billion square metres, and the total energy consumption of building operation was 1.09 billion tce (The unit tce is short for ton of standard coal equivalent. One tce is equivalent to 29.271 MJ/kg × 1000 kg = 29.271 GW), accounting for approximately 22% of the total energy consumption in China^33^. The energy consumption per square metre of building operation was approximately 18.14 kgce.

The operation and maintenance costs of house building include property management fees and house maintenance fees, which vary greatly in different regions and types of buildings. In this paper, the property management fee of small high-rise ordinary residences is taken as the calculated value, that is, 1.5 yuan per square metre per month. The house maintenance fee is calculated according to the interest of the maintenance fund, that is, 2.4 yuan per square metre per year; in this case, the annual operation and maintenance cost is 21 yuan per square metre. The average annual emissions of CO_2_, SO_2_, CO and NO_x_ per square building area in the operation and maintenance stage are 0.105 t, 0.5 kg, 0.09 kg and 0.34 kg, respectively^32^.

### Demolition and disposal stage

The life end stage includes building demolition and construction waste disposal. The total area of demolished buildings in China is approximately 1.5 billion square metres every year^33^. According to the field investigation, based on house buildings with different structural systems, heights and locations, the comprehensive quotation of professional demolition companies for demolishing house buildings, cleaning up construction waste, loading and transporting it to the dump is approximately 55 ~ 215 yuan per square metre, and the average price is 135 yuan per square metre. For different structural systems, the quantity and composition of construction waste during demolition are different. Based on the widely used reinforced concrete civil buildings, when one square metre of building is demolished, 1.8 tons of construction waste will be generated^34^. It takes 59.3 MJ, or 2.02 kgce, to demolish one square metre of building; at the same time, CO_2_, SO_2_, NO_X_ and other environmental impacts are discharged, which are 3.7 kg, 8.0 g and 34.7 g, respectively^32^.

In 2020, the comprehensive utilization rate of construction waste in China reached 50%^35^. Different disposal methods of construction waste have different disposal costs and environmental impacts^36^. Therefore, the disposal cost of construction waste and the emission of environmental impacts can be calculated (Appendix Table 2). The resource consumption and environmental impact emissions of demolishing and disposing of one square metre of building can be obtained (Table 4).

**Table 4:** Resource consumption and environmental impact emissions per square metre area in the life end stage.

Item	Cost /yuan	Energy/tce	CO_2_/t	SO_2_/t	CO/t	NO_x_/t	H_2_S/t	Pb/t	Cd/t	Cr/t	Hg/t	Dust/t
Quantity	1.54E + 02	4.62E − 03	7.33E − 02	5.43E − 04	6.84E − 04	4.22E − 05	6.66E − 04	3.64E − 05	7.88E − 06	3.11E − 06	3.38E − 07	1.78E − 03

## Result and analysis

### Construction EF

Based on the average material consumption and environmental impact emissions, the average emergy footprint per square metre of house building in the construction stage is 1.15 × 10^−3^ hm^2^ (Table 5). The transformity comes from Ref.^17^, and is similar hereinafter. According to Eq. (5) and Table 5, calculation of the the transformity of house buildings results in 8.92 × 10^17^ sej/hm^2^.year, assuming that the average service life of the house building is 50 years.

**Table 5:** Emergy footprint per square metre of house building in the construction stage.

Factors	Unit	Quantity	Transformity (sej/unit)	Emergy (sej)	Emergy footprint (hm^2^)	Proportion (%)
Steel	t	4.78E − 02	5.51E + 15	2.63E + 14	3.34E − 04	29.20
Cement	t	1.43E − 01	1.74E + 15	2.49E + 14	3.16E − 04	27.63
Sand & Gravel	t	5.94E − 01	1.63E + 14	9.68E + 13	1.23E − 04	10.74
Aluminium	t	1.08E − 02	1.30E + 16	1.41E + 14	1.79E − 04	15.60
Wood	m^3^	2.21E − 02	3.32E + 14	7.34E + 12	9.33E − 06	0.81
Glass	t	7.05E − 04	7.90E + 15	5.57E + 12	7.08E − 06	0.62
Energy	tce	4.25E − 03	1.17E + 15	4.98E + 12	6.32E − 06	0.55
Employed staff	Person. year	3.05E − 03	4.71E + 15	1.44E + 13	1.83E − 05	1.60
Water	t	1.11E + 00	1.60E + 12	1.78E + 12	2.26E − 06	0.20
Brick	t	1.06E − 01	3.80E + 14	4.04E + 13	5.13E − 05	4.48
Ceramic	t	1.90E − 02	3.80E + 14	7.23E + 12	9.19E − 06	0.80
COD	t	3.86E − 04	7.90E + 15	3.05E + 12	3.87E − 06	0.34
NH_3_-N	t	2.51E − 06	9.88E + 15	2.48E + 10	3.16E − 08	0.00
SO_2_	t	6.12E − 04	1.64E + 15	1.00E + 12	1.28E − 06	0.11
NOx	t	6.21E − 04	1.64E + 15	1.02E + 12	1.29E − 06	0.11
CO_2_	t	8.07E − 02	2.30E + 14	1.86E + 13	2.36E − 05	2.06
CO	t	3.33E − 03	9.34E + 13	3.11E + 11	3.95E − 07	0.03
Floor space	m^2^.year	1.60E + 00	2.88E + 13	4.61E + 13	5.86E − 05	5.11
Total				9.01E + 14	1.15E − 03	100

**Figure 3:**
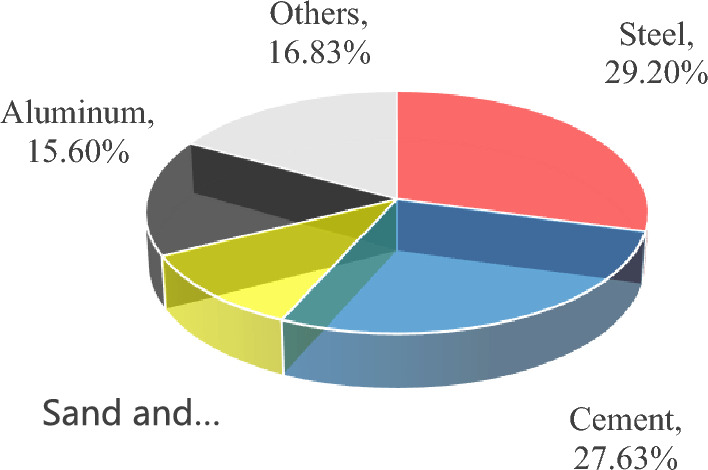
Emergy footprint composition proportion of house buildings in the construction stage.

The consumed building materials, such as steel, cement, aluminium, sand and gravel, are the main aspects of its emergy footprint, which are 3.34 × 10^−4^ hm^2^ (29.20%), 3.16 × 10^−4^ hm^2^ (27.63%), 1.79 × 10^−4^ hm^2^ (15.60%) and 1.23 × 10^−4^ hm^2^ (10.74%), respectively, as shown in Fig. 3. The emergy footprint of steel and cement consumption accounts for 56.83% of the total emergy footprint in the construction stage. By optimizing the building design scheme, the building structure system and component size can be optimized^37^. Optimizing the design scheme, scientifically organizing the construction and controlling the consumption of steel and cement are the main ways to reduce the emergy footprint of house buildings in the construction stage.

**Figure 4:**
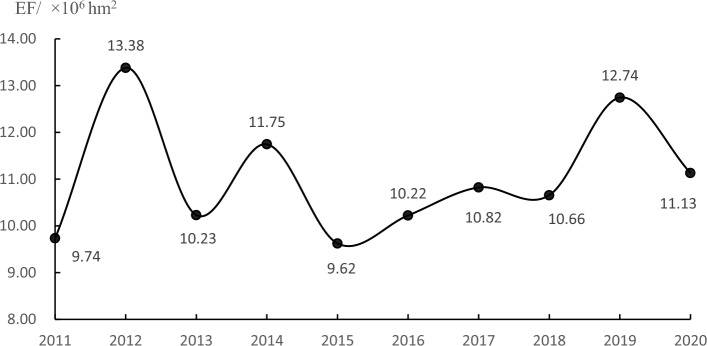
Emergy footprint of house buildings in the construction stage (2011–2020).

The housing sector in China has developed rapidly in the past ten years, and the annual construction area has rapidly increased from 8.04 × 10^9^ m^2^ in 2011 to 1.39 × 10^10^ m^2^ in 2020, with an average annual increase of 5.62%. In the past twenty years, especially in the last ten years, China has made rapid progress in building technology, and the level of construction management has been continuously improved. Although the annual construction area of house buildings has increased rapidly in the past ten years, the total emergy footprint of house buildings in the construction stage has fluctuated and declined (Fig. 4). The rise and fall reflect that the comprehensive efficiency of China's housing sector in society, economy and environment is improving.

**Figure 5:**
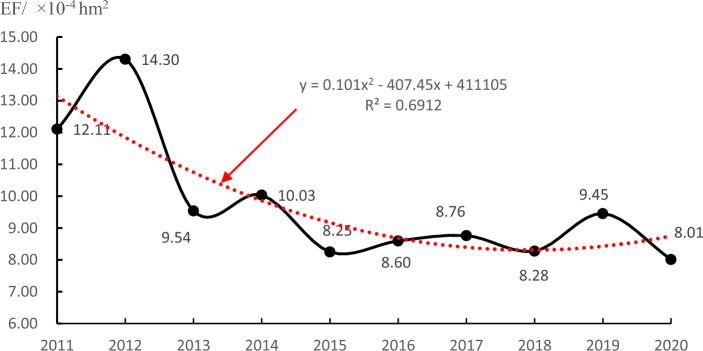
Emergy footprint per square metre of house building in the construction stage (2011–2020).

In the construction stage of house buildings in China, the emergy footprint per square metre area has generally shown a shock downwards trend in the past decade, from 12.11 × 10^–4^ hm^2^ in 2011 to 8.01 × 10^–4^ hm^2^ in 2020 (Fig. 5). This shows that the impact of per square metre house building in China on the social and economic environment is gradually decreasing in the construction stage with the progress of China's building materials science and technology, as well as the improvement of construction technology and management level.

**Figure 6:**
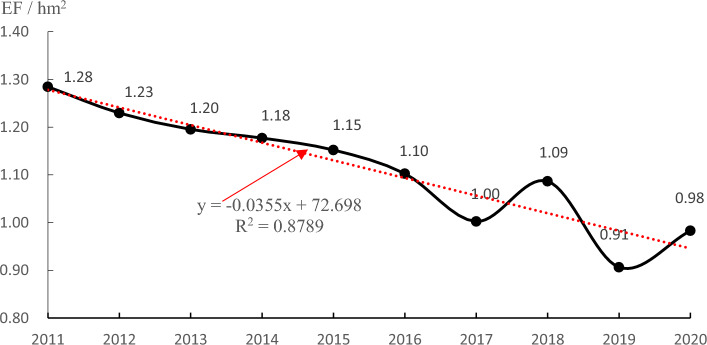
Emergy footprint per million yuan output value of house buildings (2011–2020).

The emergy footprint per million yuan output value of the house building sector in China shows a downwards trend, from 1.28 hm^2^ in 2011 to 0.98 hm^2^ in 2020 (Fig. 6), which shows the improving eco-efficiency of the house building sector in China. This result shows that the ecological impact caused by the same million yuan of output value is declining; in other words, the same ecological impact will bring more output value. There are two reasons for this: one is that the industrial structure of the housing construction industry has changed, and there are more and higher value-added industries; the other is perhaps that the utilization efficiency of building materials is improving with the development of the industry, and the waste phenomenon is curbed.

### Operation and maintenance EF

**Figure 7:**
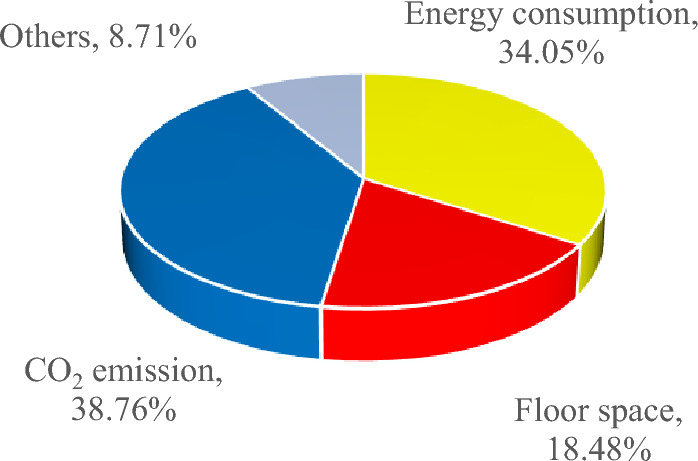
Emergy footprint composition proportion of house buildings in the use stage.

The annual emergy footprint of operation and maintenance per square metre of house building is 7.92 × 10^–5^ hm^2^ (Appendix Table 3), in which the emergy footprint of operation energy consumption, CO_2_ emissions and floor space account for 34.05%, 38.76% and 18.48%, respectively (Fig. 7). Green ecological buildings are conducive to reducing the operation energy consumption of buildings^38^. Popularizing green star-rated buildings and reducing the energy consumption and CO_2_ emissions of house buildings are the main measures to reduce the emergy footprint in the operation and maintenance stage. The total house building in China is 60.1 billion m^2^, and the total footprint of 1 year of operation and maintenance reaches 4.76 × 10^6^ hm^2^.

### Demolition and disposal EF

The emergy footprint of the resource consumption and environmental impact emissions of the demolished per square metre building is 3.73 × 10^−5^ hm^2^ (Appendix Table 4). Among them, the emergy footprint of the cost of demolition accounts for an absolute proportion, amounting to 88.81%. Approximately 1.50 × 10^−9^ m^2^ of buildings are demolished in China every year, and the emergy footprint reaches 5.59 × 10^4^ hm^2^ every year.

Construction waste disposal is divided into comprehensive utilization and direct landfill. The emergy footprint per square metre demolished building with the comprehensive utilization method is 3.10 × 10^−5^ hm^2^ (Appendix Table 5). The three main factors that affect the social environment by comprehensive utilization are CO_2_ mission, energy consumption and cost, which are 7.73 × 10^−6^ hm^2^ (24.93%), 7.46 × 10^−6^ (24.04%) and 4.37 × 10^−6^ hm^2^ (14.09%), respectively.

The energy consumption and environmental impact of construction waste generated per square metre demolished building with the direct landfill method is 1.16 × 10^−4^ hm^2^ (Appendix Table 6). The three main factors that affect the social environment by the direct landfill method are CO_2,_ Cd and Pb which are discharged into the environment, and they amount to 3.29 × 10^−5^ hm^2^ (28.36%), 2.94 × 10^−5^ hm^2^ (25.30%) and 2.70 × 10^−5^ hm^2^ (23.22%), respectively.

The emergy footprint of direct landfill of construction waste is 3.74 times that of comprehensive utilization. Among them, the emergy footprint of heavy metals such as Cd and CO_2_ discharged into the environment by direct landfill is 12.6 times and 4.26 times that by comprehensive utilization, respectively. Therefore, improving the comprehensive utilization rate of construction waste is an effective way to reduce the negative impact of construction waste on the environment. According to the current comprehensive utilization ratio of construction waste in China (50%), the average emergy footprint per square metre of house building is 7.36 × 10^−5^ hm^2^. Approximately 1.50 × 10^9^ m^2^ of buildings are demolished in China every year, and the resulting emergy footprint is 1.10 × 10^5^ hm^2^.

**Figure 8:**
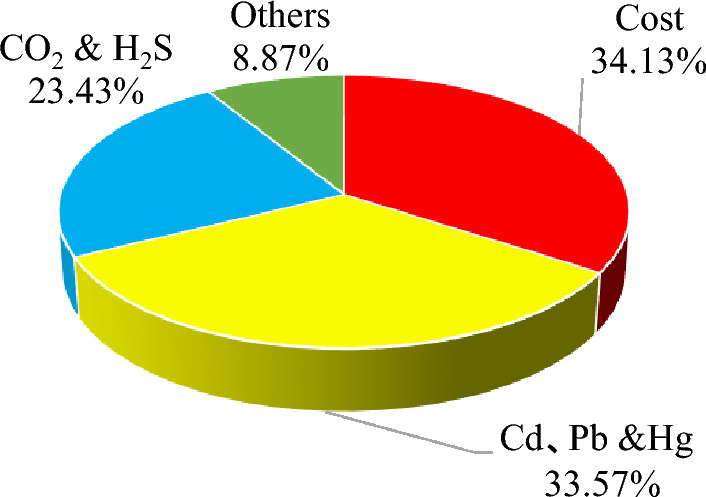
Emergy footprint composition proportion of house buildings in demolition and disposal stage.

The emergy footprint of the resource consumption and environmental impact of the demolition and disposal per square metre building is 1.11 × 10^−4^ hm^2^ (Appendix Table 7), of which the emergy footprint of the demolition cost accounts for 34.13%, the emergy footprint of heavy metals such as Cd, Pb and Hg accounts for 33.57%, and the emergy footprint of CO_2_ and H_2_S accounts for 23.43%. The proportion of the emergy footprint in the demolition and disposal stage is shown in Fig. 8.

### Life cycle EF

During the life cycle, house buildings go through the stages of construction, operation and maintenance, and demolition and disposal. After the house building is built, the emergy footprint of construction is a fixed value. Under the condition of maintaining the existing methods of demolition and construction waste disposal, the emergy footprint of demolition and disposal is a constant value. In the operation and maintenance stage, the energy consumption of buildings and the emission of environmental impacts such as CO_2_ will increase with the increase in operation and maintenance years.

**Figure 9:**
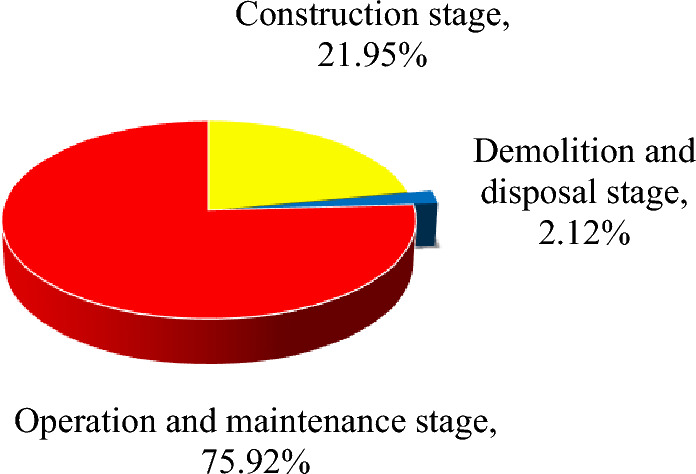
Emergy footprint composition proportion of house buildings during the life cycle.

The design service life of most house buildings in China is 50 years. Starting from the date when the house building is completed and accepted, assuming that it will be operated for 50 years, the emergy footprint of the house building life cycle per square metre is 5.22 × 10^–3^ hm^2^. The emergy footprint in the construction stage is 1.15 × 10^–3^ hm^2^ (accounting for 21.95%); the emergy footprint in the use stage is 3.96 × 10^–3^ hm^2^ (75.92%). The emergy footprint of the life end stage is small, and is only 1.11 × 10^–4^ hm^2^ (2.12%), as shown in Fig. 9.

Unless there is a great improvement in the production process of building materials and progress in construction technology, there is little space to reduce the emergy footprint in the construction stage. Therefore, reducing the emergy footprint in the operation and maintenance period of house buildings is an important way to reduce the emergy footprint in the life cycle of house buildings. The emergy footprint occupation intensity per square metre of house building in the construction stage (3.82 × 10^–4^ hm^2^/yr) is 4.82 times that in the use stage (7.92 × 10^–5^ hm2/yr). It is important to reduce the impact of house building construction activities to decrease the emergy footprint occupation in the construction stage.

### Ecological profit and loss analysis

The emergy capacity will be provided after the completion of the house building is accepted and handed over. For every square metre of house building, the annual emergy capacity is 1.13 × 10^–3^ hm^2^. From the perspective of the life cycle, the emergy footprint of the newly completed house building remains unchanged in the construction, demolition and disposal stages.

Under the condition of keeping the average operation and maintenance cost and energy consumption unchanged, the annual emergy footprint of the operation and maintenance stage is 6.46 × 10^5^ hm^2^, and the emergy footprint of operation and maintenance increases with increasing operation and maintenance years. Taking 2020 as an example, the emergy footprint of construction is 9.72 × 10^6^ hm^2^, that of demolition and disposal is 3.55 × 10^5^ hm^2^ and that of newly completed house buildings is 3.75 × 10^5^ hm^2^; that is, the supply of new emergy capacity is 3.75 × 10^5^ hm^2^ per year. According to the emergy footprint and emergy capacity data in the life cycle of the house buildings, the ecological impact coefficient of the corresponding year can be calculated (Appendix Table 8).

**Figure 10:**
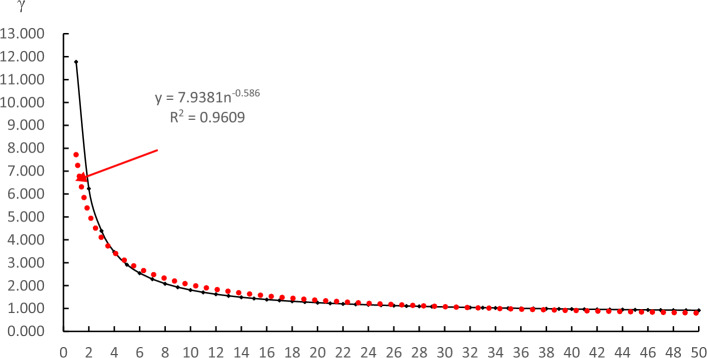
The relationship between the ecological impact coefficient of house buildings and their service life.

According to the service life and ecological impact coefficient of the house buildings, the simulation fitting is carried out, and the red dotted line is the simulation fitting curve, as shown in Fig. 10. The ecological impact coefficient of house buildings decreases with increasing service life. Extending the service life of house buildings can effectively reduce its ecological impact coefficient. At the same time, the empirical formula of the ecological impact coefficient of the comprehensive impact of house buildings on society, economy and environment can be obtained.

8 $$\upgamma =7.9381{\times n}^{-0.586}.$$

The formula shows that the ecological impact coefficient (γ) of the house buildings has a negative exponential relationship with its service life (*n*). The longer the service life is, the smaller the ecological impact coefficient is.

After the completion acceptance and hand over, the building is put into use, it will then take 36.73 years to achieve the ecological break-even. Assume that the building life cycle is 50 years and that the ecological surplus period is 13.27 years. When the service life of the house building is more than 44 years and the ecological impact coefficient of the house building is less than 0.95, the impact of the house building on society, the economy and the environment is positive (Grade I). When the service life of the house building is not less than 37 years, the ecological impact coefficient of the house building is less than 1, which is more favourable to society, the economy and the environment (Grade II). If the service life of the house building is more than 9 years but less than 37 years after completion, the impact of the house building on society, the economy and the environment is unfavourable (Grade IV); if the service life of the house building is less than 9 years after completion, the impact of the house building on society, the economy and the environment is extremely unfavourable (Grade V).

## Discussion and conclusion

The environmental impact of house buildings during the life cycle has been widely concerned. The purpose of this paper is to quantitatively and uniformly evaluate the multi-factor comprehensive impact of social-economic-environmental complex system in the house buildings life cycle.

First, based on emergy footprint theory and LCA theory, this study defines the concepts of the emergy footprint occupancy, carrying capacity supply, ecological impact coefficient and ecological impact level and proposes the emergy footprint model for multifactor quantitative analysis of house buildings, which can comprehensively help implement the life cycle quantitative impact assessment of house buildings on society, the economy and the environment. This model can quantitatively evaluate the social, economic and environmental impact of a single building or a certain type of building and can also make a comprehensive comparative analysis of the impact of the different types or different structural systems of house buildings on society, economy and environment.

Second, optimizing the design scheme, scientifically organizing the construction and controlling the consumption of steel and cement are the main ways to reduce the emergy footprint of house buildings in the construction stage. By promoting green buildings, reducing energy consumption and carbon dioxide emissions are the main measures in the use stage. Improving the comprehensive utilization rate of construction waste can effectively reduce the negative impact of construction waste on the environment.

Third, both the per square metre and per million yuan output value of the house building emergy footprint have shown decreasing trends in the last ten years, which shows that the social, ecological and environmental efficiency of house buildings in China is improving. In the life cycle of house buildings, the emergy footprint in the operation and maintenance stage is the largest (75.92%), followed by the construction stage, and the life end stage is the smallest (2.12%), but the emergy footprint occupation intensity per square metre of house building in the construction stage is 4.82 times that in the operation and maintenance stage. Reducing energy consumption and carbon dioxide emissions in the operation and maintenance stage are the cruxes to reducing the life cycle emergy footprint of house buildings. The literature analyzes the ecological footprint of a green building in its life cycle, and the ecological footprint in the operation and maintenance stage occupies the largest (73.27%)^39^, which is consistent with the conclusion of this study. The model constructed in this paper is applicable and reliable. As a key parameter in the ecological footprint model, the equilibrium factor is different in different research institutions and the same research institution in different years^17^, it affects the comparability of research results to some extent. Through the relatively stable transformity, the emergy footprint model can convert multiple influencing factors with different properties and dimensions into emergy, thus making the research results more comparable.

Fourth, the empirical formula of the relationship between the ecological impact coefficient and service life in the life cycle of house buildings is obtained for the first time. The formula is helpful for related researchers to quickly estimate the ecological impact coefficient through the service life of the building, so as to judge the environmental impact grade of the building. Under the condition of keeping the operation and maintenance cost and energy consumption unchanged, the ecological impact coefficient of the house buildings has a negative exponential relationship with its service life. The longer the service life is, the smaller the ecological impact coefficient is. When the length of house building service is 36.73 years, it just reaches the ecological break-even; when the length of service is more than 44 years, the impact level is grade I, which is a positive impact for the building on society, the economy and the environment. If it is demolished in less than nine years after its completion, the impact of the building on society, the economy and the environment will be extremely unfavourable, namely, the ecological impact level is grade V.

Finally, the comprehensive impact of house buildings on society, the economy and the environment is graded (see Table 1) in this paper. Because there is no other literature about emergy footprint model of house buildings, the scientific rationality of the classification needs further study.

### Supplementary Information

Supplementary Tables.

## Data Availability

All data generated or analysed during this study are included in this published article (and its supplementary information files).
