# Supplementary material for: Ecological effect life cycle assessment of house buildings based on emergy footprint model
Source: Sci Rep. 2023 Sep 30;13:16441. doi: 10.1038/s41598-023-43501-3 (PMC10543385; doi:10.1038/s41598-023-43501-3)
Supplement: Supplementary file 1 — Supplementary Tables. [file 41598_2023_43501_MOESM1_ESM.pdf]

Appendix

Appendix Table 1 China's construction output value, building area and main resource consumption

Item	Unit	2011	2012	2013	2014	2015	2016	2017	2018	2019	2020
GDP	yuan	4.84E+13	5.39E+13	5.96E+13	6.47E+13	6.92E+13	7.46E+13	8.29E+13	9.16E+13	9.95E+13	1.03E+14
Output value of construction	yuan	1.17E+13	1.37E+13	1.60E+13	1.77E+13	1.81E+13	1.94E+13	2.14E+13	2.26E+13	2.69E+13	2.64E+13
#House buildings	yuan	7.17E+12	8.71E+12	1.03E+13	1.14E+13	1.16E+13	1.24E+13	1.41E+13	1.36E+13	1.70E+13	1.62E+13
Proportion of house buildings	%	61.23	63.50	64.07	64.44	64.15	63.82	65.96	60.09	63.37	61.33
Construction area	㎡	8.52E+09	9.86E+09	1.13E+10	1.25E+10	1.24E+10	1.26E+10	1.32E+10	1.37E+10	1.44E+10	1.49E+10
#House buildings	㎡	8.04E+09	9.36E+09	1.07E+10	1.17E+10	1.17E+10	1.19E+10	1.24E+10	1.29E+10	1.35E+10	1.39E+10
Completed area	㎡	3.16E+09	3.59E+09	4.02E+09	4.23E+09	4.21E+09	4.22E+09	4.19E+09	4.11E+09	4.02E+09	3.85E+09
#House buildings	㎡	2.94E+09	3.39E+09	3.80E+09	4.00E+09	3.96E+09	3.97E+09	3.94E+09	3.84E+09	3.75E+09	3.56E+09
Steel	t	6.63E+08	9.15E+08	7.43E+08	9.44E+08	7.68E+08	8.46E+08	8.44E+08	8.94E+08	1.07E+09	9.89E+08
Cement	t	2.84E+09	3.73E+09	2.40E+09	2.61E+09	2.00E+09	2.13E+09	2.25E+09	2.42E+09	2.43E+09	2.32E+09
Wood	t	2.37E+08	3.91E+08	3.02E+08	3.79E+08	3.64E+08	4.49E+08	4.49E+08	4.87E+08	4.92E+08	5.39E+08
Glass	t	6.91E+06	1.24E+07	3.09E+07	1.47E+07	9.55E+06	9.24E+06	1.04E+07	1.09E+07	1.13E+07	9.33E+06
Aluminium	t	3.83E+07	6.38E+07	5.16E+07	6.05E+07	5.63E+07	5.58E+07	5.45E+07	6.38E+07	1.30E+08	6.17E+07
Energy	tce	5.87E+07	6.17E+07	7.02E+07	7.52E+07	7.70E+07	7.99E+07	8.56E+07	8.69E+07	9.14E+07	9.32E+07
Employed staff	person	5.02E+07	4.63E+07	5.03E+07	5.56E+07	5.58E+07	5.74E+07	6.15E+07	5.95E+07	6.22E+07	5.37E+07

Appendix Table 2 Resource consumption and environmental impacts of disposal construction waste from per square meter demolished building

Disposal method	Cost /yuan	Energy /MJ	CO_2 /t	SO_2 /t	CO /t	NO_x /t	H_2S /t	Pb /t	Cd /t	Cr /t	Hg /t	Dust /t
Comprehensive utilization	1.78E+1	5.02E-3	2.65E-2	1.07E-3	1.15E-3	7.74E-6	4.14E-4	5.65E-6	1.12E-6	4.50E-7	4.81E-8	1.33E-3
Direct landfill	2.07E+1	1.87E-4	1.13E-1	4.32E-7	2.16E-4	7.24E-6	9.18E-4	6.71E-5	1.46E-5	5.76E-6	6.28E-7	2.23E-3
Average	1.93E+1	2.60E-3	6.96E-2	5.35E-4	6.84E-4	7.49E-6	6.66E-4	3.64E-5	7.88E-6	3.11E-6	3.38E-7	1.78E-3

Appendix Table 3 Emergy footprint of operation and maintenance for per square meter house building each year

Factors	Unit	Quantity	Transformiy (sej/unit)	Emergy (sej)	Emergy footprint (hm^2)	Proportion (%)
Cost	yuan	2.10E+01	1.93E+11	4.05E+12	5.15E-06	6.50
Energy	tce	1.81E-02	1.17E+15	2.12E+13	2.70E-05	34.05
Floor space	m^2.yr	4.00E-01	2.88E+13	1.15E+13	1.46E-05	18.48
CO_2	t	1.05E-01	2.3E+14	2.42E+13	3.07E-05	38.76
SO_2	t	5.00E-04	1.64E+15	8.20E+11	1.04E-06	1.32
NO_X	t	3.40E-04	1.64E+15	5.58E+11	7.09E-07	0.89
Total				6.23E+13	7.92E-05	100

Appendix Table 4 Emergy footprint of per square meter demolition building

Factors	Unit	Quantity	Transformiy (sej/unit)	Emergy (sej)	Emergy footprint (hm^2)	Proportion (%)
Cost	yuan	1.35E+02	1.93E+11	2.61E+13	3.31E-05	88.81
Energy	tce	2.02E-03	1.17E+15	2.36E+12	3.00E-06	8.06
CO_2	t	3.70E-03	2.30E+14	8.51E+11	1.08E-06	2.90
SO_2	t	8.00E-06	1.64E+15	1.31E+10	1.67E-08	0.04
NOx	t	3.47E-05	1.64E+15	5.69E+10	7.23E-08	0.19
Total				2.93E+13	3.73E-05	100

Appendix Table 5 Emergy footprint of per square meter demolished building with comprehensive utilization method

Factors	Unit	Quantity	Transformiy (sej/unit)	Emergy (sej)	Emergy footprint (hm^2)	Proportion (%)
Cost	yuan	1.78E+01	1.93E+11	3.44E+12	4.37E-06	14.09
Energy	tce	5.02E-03	1.17E+15	5.87E+12	7.46E-06	24.04
CO_2	t	2.65E-02	2.30E+14	6.09E+12	7.73E-06	24.93
SO_2	t	1.07E-03	1.64E+15	1.75E+12	2.23E-06	7.18
CO	t	1.15E-03	9.34E+13	1.08E+11	1.37E-07	0.44
NO_x	t	7.74E-06	1.64E+15	1.27E+10	1.61E-08	0.05
H_2S	t	4.14E-04	5.38E+15	2.23E+12	2.83E-06	9.12
Pb	t	5.65E-06	3.16E+17	1.79E+12	2.27E-06	7.32
Cd	t	1.12E-06	1.58E+18	1.76E+12	2.24E-06	7.22
Cr	t	4.50E-07	1.98E+17	8.91E+10	1.13E-07	0.36
Hg	t	4.81E-08	1.58E+19	7.59E+11	9.65E-07	3.11
Dust	t	1.33E-03	3.90E+14	5.19E+11	6.60E-07	2.13
Total				2.44E+13	3.10E-05	100

Appendix Table 6 Emergy footprint of per square meter demolished building with direct landfill method

Factors	Unit	Quantity	Transformiy (sej/unit)	Emergy (sej)	Emergy footprint (hm^2)	Proportion (%)
Cost	yuan	2.07E+01	1.93E+11	4.00E+12	5.08E-06	4.37
Energy	tce	1.87E-04	1.17E+15	2.19E+11	2.79E-07	0.24
CO_2	t	1.13E-01	2.30E+14	2.59E+13	3.29E-05	28.36
SO_2	t	4.32E-07	1.64E+15	7.08E+08	9.00E-10	0.00
CO	t	2.16E-04	9.34E+13	2.02E+10	2.56E-08	0.02
NO_x	t	7.24E-06	1.64E+15	1.19E+10	1.51E-08	0.01
H_2S	t	9.18E-04	5.38E+15	4.94E+12	6.28E-06	5.40
Pb	t	6.71E-05	3.16E+17	2.12E+13	2.70E-05	23.22
Cd	t	1.46E-05	1.58E+18	2.31E+13	2.94E-05	25.30
Cr	t	5.76E-06	1.98E+17	1.14E+12	1.45E-06	1.25
Hg	t	6.28E-07	1.58E+19	9.93E+12	1.26E-05	10.86
Dust	t	2.23E-03	3.90E+14	8.70E+11	1.11E-06	0.95
Total				9.14E+13	1.16E-04	100

Appendix Table 7 Emergy footprint of per square meter demolition and disposal house building

Factors	Unit	Quantity	Transformiy (sej/unit)	Emergy (sej)	Emergy footprint (hm^2)	Proportion (%)
Cost	yuan	1.54E+02	1.93E+11	2.98E+13	3.78E-05	34.13
Energy	tce	4.62E-03	1.17E+15	5.41E+12	6.87E-06	6.20
CO_2	t	7.33E-02	2.30E+14	1.69E+13	2.14E-05	19.32
SO_2	t	5.43E-04	1.64E+15	8.90E+11	1.13E-06	1.02
CO	t	6.84E-04	9.34E+13	6.39E+10	8.12E-08	0.07
NO_x	t	4.22E-05	1.64E+15	6.92E+10	8.79E-08	0.08
H_2S	t	6.66E-04	5.38E+15	3.58E+12	4.55E-06	4.11
Pb	t	3.64E-05	3.16E+17	1.15E+13	1.46E-05	13.18
Cd	t	7.88E-06	1.58E+18	1.24E+13	1.58E-05	14.26
Cr	t	3.11E-06	1.98E+17	6.15E+11	7.81E-07	0.70
Hg	t	3.38E-07	1.58E+19	5.34E+12	6.79E-06	6.12
Dust	t	1.78E-03	3.90E+14	6.95E+11	8.83E-07	0.80
Total				8.72E+13	1.11E-04	100

Appendix Table 8 Service life and ecological impact coefficient of house buildings

Year	0	1	2	3	4	5	6	7	8	9
0	∞	11.775	6.236	4.390	3.467	2.914	2.544	2.281	2.083	1.929
1	1.806	1.705	1.621	1.550	1.490	1.437	1.391	1.350	1.314	1.281
2	1.252	1.226	1.202	1.180	1.160	1.142	1.124	1.109	1.094	1.080
3	1.068	1.056	1.045	1.034	1.024	1.015	1.006	0.998	0.990	0.982
4	0.975	0.969	0.962	0.956	0.950	0.945	0.939	0.934	0.929	0.925
5	0.920	0.916	0.911	0.907	0.904	0.900	0.896	0.893	0.889	0.886
6	0.883	0.880	0.877	0.874	0.872	0.869	0.866	0.864	0.861	0.859
7	0.857	0.854	0.852	0.850	0.848	0.846	0.844	0.842	0.840	0.839
8	0.837	0.835	0.834	0.832	0.830	0.829	0.827	0.826	0.824	0.823
9	0.822	0.820	0.819	0.818	0.816	0.815	0.814	0.813	0.812	0.810
